# Supplementary material for: Ultrafast non-excitonic valley Hall effect in MoS2/WTe2 heterobilayers
Source: Nat Commun. 2021 Mar 12;12:1635. doi: 10.1038/s41467-021-21013-w (PMC7955044; doi:10.1038/s41467-021-21013-w)
Supplement: Supplementary file 1 — Supplementary Information [file 41467_2021_21013_MOESM1_ESM.pdf]

Supplementary Information for

Ultrafast non-excitonic valley Hall effect in MoS_2/WTe_2 heterobilayers

Jekwan Lee[1,2], Wonhyeok Heo[2], Myungjun Cha[1,2], Kenji Watanabe[3], Takashi Taniguchi[3], Jehyun Kim[1], Soonyoung Cha[4], Dohun Kim[1], Moon-Ho Jo[4], and Hyunyong Choi[1,2]

[1]*Department of Physics and Astronomy, Seoul National University, Seoul 08826, Korea*

[2]*Institute of Applied Physics, Seoul National University, Seoul 08826, Korea*

[3]*Advanced Materials Laboratory, National Institute for Materials Science, 1-1 Namiki, Tsukuba 305-0044, Japan*

[4] *Center for Artificial Low Dimensional Electronic Systems, Institute for Basic Science, Pohang 37673, Korea*

Contents

Supplementary Notes

1. Sample preparation 2
2. Experimental setup 4
3. Additional evidence for the injection of spin-polarized electrons 7
4. The suppressed exciton photoluminescence 9
5. Extended data sets from the TRKR measurements 12
6. Hall mobility calculation 13
7. Theoretical analysis based on the Kubo formula 14
8. Gate voltage dependence of the valley Hall velocity 16

Supplementary Table 17

Supplementary Figures 18

Supplementary References 32

Supplementary Note 1 - Sample preparation

The bulk single crystal of MoS_2 and WTe_2 (HQ graphene^{TM}), and the high-quality hexagonal boron nitride (hBN) are used to obtain the monolayers of MoS_2, WTe_2, and thin-film hBN by mechanical exfoliation. For the monolayer WTe_2 crystal, prior studies [1,2] have shown that it becomes the quantum spin Hall phase up to 100 K when thinned down to a monolayer, and the crystal structure changes from the T_d phase in the bulk limit to the 1T′ phase in the monolayer limit. Following these recipes, the monolayer flake of 1T′-WTe_2 was cleaved from the bulk T_d-WTe_2 single crystal, and the experiments were performed at 78 K using the 1T′ phase WTe_2. For the hBN crystals, they were directly imported from the National Institute of Materials Science (NIMS), Japan. We have used the exfoliated thin hBN layers to encapsulate the whole device. The MoS_2 and WTe_2 monolayer flakes were isolated from the bulk crystal on polydimethylsiloxane (PDMS) by using thermal released tape. The exfoliated monolayer flakes were then transferred onto a 300 nm Si/SiO_2 substrate by the PDMS viscoelastic stamping technique [3].

In our device, we have used the Au bottom gate to induce a large electrostatic potential in the MoS_2/WTe_2 channel. For the device fabrication, the bottom gate electrode is patterned on a Si/SiO_2 substrate by the standard electron beam lithography, followed by the thermal evaporation of Ti (5nm) and Au (110nm). A thin hBN (15 nm) flake was used as a gate insulator, which was transferred using the PDMS stamp. The 5 nm thick Pd electrodes were then deposited onto the gate insulator to apply the electrical bias along the longitudinal direction. Using a thin film of polycarbonate (PC, Sigma Aldrich, 6% dissolved in 1.0% ethanol stabilized chloroform, Sigma Aldrich), we sequentially picked up the hBN layer (top encapsulation, 20 nm), monolayer MoS_2, and monolayer WTe_2. The stacked heterostructure was transferred onto

the pre-patterned electrodes. During the ‘pick up and transfer’ process, we have used a stepping piezo-electric stage (MAX312D/M, Thorlabs™) to stack layers with a speed of 50 nm/s. We also have equipped a hot chuck to supply a steady-state heat flow at a rate of 0.1 °C per minute. The fabrication process was performed in an inert atmosphere, provided by a nitrogen-filled glove box (H_2O < 0.7 ppm, O_2 < 0.7 ppm), to prevent the degradation of WTe_2 from the oxygen and the water vapor.

Finally, the completed device was sent to the post-annealing process (200 °C for 10 hours under Ar flow of 200 ccm and H_2 flow of 50 ccm) to eliminate any organic resides and to achieve a sufficiently good interfacial contact. The electrical characteristics of the complete device are shown in Supplementary Fig. 1.

Supplementary Note 2 - Experimental setup

2-1. Experimental setup

For the remote ultrashort-pulse excitation and the ultrafast Kerr rotation measurements, we have used the Coherent RegA (model 9050), which generates a 50-fs, 1.55 eV pulse at a 250 kHz repetition rate. A beamsplitter (70:30) is used to obtain the synchronized pump and probe pulses for the time-resolved experiments. The probe pulse with the energy of 1.88 eV was prepared through the white-light generation by injecting a portion of 1.55 eV pulse (70%) into a sapphire crystal. To measure the spatially resolved PL, a continuous wave (c.w.) 532 nm (2.33 eV) diode laser was used. The sample was mounted in a liquid nitrogen-cooled optical cryostat (Hi-Res, Oxford™), and the electrical contacts were made using Indium wires between the optical cryostat and the electrodes of the sample. All experiments were performed at the temperature of 78±0.1 K. To reduce the GVD, and the setup was equipped with a reflective objective lens. The minimum spot size was about 2 μm. The spatially resolved experiment was performed via the dual-axis scanning galvo mirror (GVS012, Thorlabs™).

2-2. Spatially resolved differential photoluminescence measurements

Supplementary Fig. 2 shows the schematics of the spatially resolved differential PL measurements. The differential PL (the PL intensity difference with/without the remote pump excitation) was used to measure the VHE in MoS_2, where the excess spin-polarized electrons are injected from the WTe_2 layer to the MoS_2 layer. The charge injection was verified in the following way. First, the remote pump was positioned at the edge of the WTe_2 layer; the photon energy (1.55 eV) is well below the bandgap of the MoS_2 monolayer, i.e. 1.88 eV (660 nm).

Then, the remote pump was modulated by a mechanical chopper with a chopping frequency of 700 Hz, and the synchronized PL signal obtained by spatially scanning the 532 nm (2.33 eV) laser. The intensity of the circularly polarized remote pump was 278 W/cm^2 and that of the circularly polarized (same helicity with the remote pump) 532 nm c.w. scanning laser was 39.8 W/cm^2.

The 2D map of the differential PL signals was obtained by combining the position information from the scanning mirror and the PL of the MoS_2 A exciton. The PL spectrum was measured by dispersing the luminescence signal through a monochromator (MonoRa320i, Dongwoo Optron^{TM}).

2-3. Ultrafast time-resolved Kerr rotation

The pump-induced Kerr rotation angle was measured in the following two ways. First, we measured the 2D map of the Kerr rotation angle in the MoS_2 layer at each pump-probe delay Δt with an applied gate voltage V_G = 2 V. The collected data provide the spatially resolved information at a given Δt. Second, when a target position is chosen, e.g. an area exhibiting the large Kerr rotation changes, we performed detailed studies of the time-resolved Kerr rotation (TRKR) with varying V_G. The corresponding data are shown in Fig. 4(a) of the main text. In both cases, the pump is far away from the MoS_2 layer, as discussed in the main text. The circularly polarized pump (1.55 eV) with the intensity of 278 W/cm^2 was mechanically chopped with the chopping frequency of 700 Hz. The linearly polarized probe (1.88 eV) with the intensity of 10 W/cm^2 was obtained after a portion of the white-light continuum was spectrally filtered. The Kerr rotation was measured using a combination of a Wollaston prism

and a balanced detector (Nirvana 2007, Newport^{TM}).

Supplementary Note 3 - Additional evidence for the injection of spin-polarized electrons

The transverse deflection of the differential PL and Kerr rotation are presented as evidence for the existence of injected electrons and the VHE in the main text. Here we present the additional evidence of the effective injection of the spin-polarized electrons and the VHE.

3-1. c.w. Kerr rotation

The c.w. laser with the center wavelength of 800 nm (1.55 eV) was prepared from the supercontinuum light source (SC-7 PROTM, YSL Photonics Co.) by using a bandpass filter with FWHM of 10 nm, and the polarization was manipulated to be linearly polarized. The wavelength of the beam was chosen to have the same value as the pulse pump in the previous TRKR experiment to achieve proper comparison.

The valley Hall deflection of relatively small valley polarization was detected under the applied longitudinal electric field, as shown in Supplementary Fig. 3. Unlike the experiments using ultrafast lasers shown in the main text, the deflection is observed throughout the entire region. In this case, the subject of the valley Hall effect is the conduction electrons that exist all over the device with no particular valley polarization. Thus, the difference between the amplitude of ultrafast Kerr rotation (main text) and c.w. Kerr measurement (here) certifies the effective injection of spin-polarized electrons.

3-2. Transverse valley Hall voltage

Similar to the method used in the early studies on the VHE [4,5], the circularly polarized c.w. light excitation is used to form the initial valley polarization, and valley Hall voltage induced in the MoS_2 channel is measured.

The measurement was performed using another device (Supplementary Fig. 4(a)) to prove the valley Hall effect induced by the c.w. light source. The bias voltage was applied between electrodes 1, 3, and electrodes 2, 4, as shown in Supplementary Fig. 4(a). The potential difference between the electrode 3 and 4 was measured by the lock-in amplifier synchronized with the light chopping frequency (c.w. 1.55 eV laser). The polarization of the c.w. pump laser was manipulated by the half-wave plate mounted on the motorized rotation stage and quarter-wave plate with a fixed axis. Supplementary Fig. 4(b) shows the valley Hall voltage by the 1.55 eV excitation at the marked point (green dot in Supplementary Fig. 4(a)) and measured at MoS_2 channel (electrode 3, 4). The apparent sign change of potential difference led by the change of pump helicity proves the effective electron injection with preserving the spin polarization.

Supplementary Note 4 - The suppressed exciton photoluminescence

In Fig. 2 of the main text, the suppression of the PL signal at the energy of 1.9 eV is used as an indicator of the injected spin-polarized electrons. The detailed mechanism of this suppression can be understood by investigating the V_G-dependent PL.

It is well known that PL from a monolayer TMD is sensitive to the carrier density. For instance, when the electron density increases as the gate voltage is applied to the monolayer TMD, the largely increased electron density results in forming negative trions. Regarding the weak oscillator strength of trions, the resultant PL signal shows a drastic decrease in exciton resonance peak while the increment of trion resonance peak is rather negligible.

Supplementary Fig. 5(a) shows the V_G-dependent PL. The total PL from the monolayer MoS_2 (black line) is decomposed into A exciton (red) and trion (blue) contributions. A significantly different V_G dependence between the exciton and trion is observed. By comparing the PL spectrum measured at the suppressed region ((0.25, 0.1) of Supplementary Fig. 5(b), right panel) with the V_G-dependent PL, we find out that the electron injection is equivalent to the increase of V_G from 2 V to 2.5 V, qualitatively (Supplementary Fig. 5(c)). Therefore, the observed PL suppression represents the effective electron injection. Furthermore, the transverse deflection of the suppressed PL reverses its spatial direction when changing the helicity of the remote pump. This is because the VHE is caused by the induced spin-polarized electrons in the transverse direction. In Supplementary Fig. 5d, we plot the helicity-resolved PL spectra when σ^+ remote pump is injected in WTe_2; note that the black line represents the MoS_2 PL without the remote pump. When the spin-polarized electron is injected by the σ^+ remote pump, the increased trion PL with the decreased exciton PL appears in the σ^+ component of PL (red line). The σ^- component of PL (blue line) is nearly the same as PL without the remote pump.

As having discussed in this note, such PL change is induced by the interlayer charge transfer (electron population transfer from WTe_2 to MoS_2) due to the remote pump excitation in WTe_2. The injected electrons are spin-polarized such that the electrons would fill the conduction band in MoS_2 whose valley index is matched with the associated electron spin, i.e. spin-valley locking. Here, if the electron spin experiences a rapid decoherence within the interlayer charge transfer time, the PL changes in MoS_2 should not exhibit a noticeable difference between the σ^+ and σ^- PL in MoS_2. In fact, the experiment result shows that this is not the case.

In Supplementary Fig. 6, the additional data of spatially resolved differential PL taken from device #1 are shown to supports our assumptions for the injection of spin-polarized electron and origin of the differential PL. When V_G = -5 V (Supplementary Fig. 6a), the 1.55 eV pump excitation in WTe_2 cannot induce the electron that would injected into MoS_2 because the Fermi level is sufficiently below the conduction band edge. Therefore, due to the type 1 heterojunction between MoS_2 and WTe_2, the difference in PL is not induced, as shown in the data. On the other hand, when V_G = 6 V, the degenerated electron doping of the MoS_2 makes the direct excitation possible by the 1.55 eV pump. The effect of the injection of electron from MoS_2 becomes negligible and thus the differential PL vanishes. Supplementary Fig. 6c shows the spatially resolved PL data with a 1.55 eV linearly polarized remote pump excitation. We have observed that the suppressed exciton PL is distributed uniformly along the edge with no noticeable valley Hall deflection. This implies the spatial distribution of K and K′ valley polarization is equally spread due to the balanced up and down spin-polarized electrons. For the zero longitudinal bias voltage, we note that although the VHE is not expected to appear in the spatially resolved differential PL, our data (Supplementary Fig. 6d, e) show that such differential PL is not completely absent. Instead, we see a signature of the VHE, though the

magnitude is very weak (about 20 % of differential PL when the bias voltage is applied). The transverse spatial displacement of the VHE is quite short (~ 0.2 μm) compared to the non-zero bias voltage (~ 0.4 μm, see Fig. 2a in the revised manuscript). Although there is no longitudinal bias voltage, the possible scenario may include the thermal gradient and thermoelectric photocurrent, which may give rise to effective potential gradient. Of course, the small magnitude of valley polarization is because the spin-polarization injection should strongly depend on the longitudinal field strength.

Supplementary Fig. 7 shows the effect of the remote pump on the PL spectrum (Supplementary Fig. 6c and Supplementary Fig. 1b are shown again for the better comparison.). Qualitatively, we see that the exciton PL with a remote pump (red color line) similarly corresponds to the PL spectrum without the remote pump when V_G is just below 3 V for both devices. Based on these observations, we can draw a conclusion that the effect of the remote pump would render the MoS_2 PL spectrum to lie at most V_G of 3 V. Here, note that although the PL data when V_G is 2 V without the remote pump is presented, this corresponds to 0 % injection efficiency because the PL with the remote pump is measured when V_G is 2 V. Therefore, we can only estimate the upper bound of the injection efficiency. For the quantitative estimation of the valley polarization injection efficiency, we use the V_G-dependent transfer data (Supplementary Fig. 7b for device #1 and Supplementary Fig. 7d for device #2). The change of electron population was inferred by calculating the ratio between the current I_{2V} and I_{3V} when V_G = 2 V and 3 V, respectively. As for device #1, I_{2V} = 0.695 nA and I_{3V} = 0.761 nA so the upper bound of efficiency $(I_{3V}-I_{2V})/I_{2V}$ is 11.2 %. Similarly, for device #2, I_{2V} = 0.876 nA and I_{3V} = 0.796 nA for device #2, thereby the upper bound of efficiency is 9.8 %.

Supplementary Note 5 - Extended data sets from the TRKR measurements

In Supplementary Fig. 8, we show the 2D Kerr rotation map when the remote pump has an opposite helicity (σ^-) compared to the main text. We see that the valley polarization experiences opposite transport direction compared to the cases of σ^+ remote pump.

The 2D data of Kerr rotation map taken at Δt = 1, 2, 5, 10, 20, 40, 70, 100 ps (V_G = 2 V) are shown in Supplementary Fig. 9. We clearly see the dynamic behavior of the transverse valley Hall transport as a function of Δt. Other than the VHE, there are some anomalies that appear in the 2D Kerr-rotation map. First, a transient negative Kerr rotation is observed at Δt = 1 ps. Because the valley polarized holes exhibit an opposite Kerr rotation angle compared to the electrons, such a negative Kerr rotation may originate from the excited hole population. The detailed physics underlying this anomaly has not been studied thoroughly because such an effect is not the major scope of this study. Second, there is a subtle local minimum and maximum located at around (-0.5, 2) μm. We regard this as an artifact caused by the sample condition, such as a localized defect (carrier trap) because this phenomenon appears at the same spatial position of (-0.5, 2) μm.

Supplementary Fig. 10 shows additional TRKR data measured at the same point where the V_G-dependent TRKR is measured in the main text, but with a much higher doping level (V_G = 6 V). Supplementary Fig. 11 shows the reference TRKR data when the pump and probe pulses are located at the same point in the monolayer MoS_2 region. The observed valley polarization lifetime of around 6.8 ps is consistent with other reported studies [6-9].

Supplementary Note 6 - Hall mobility calculation

In the main text, we estimated the Hall mobility μ_{Hall} of 4.49 x 10^3 cm^2/Vs when $V_G = 2$ V (see Fig. 3(c) in the main text), and the value was obtained based on the information presented in Supplementary Note 5. In the following, we rationalize the correlation between μ_{Hall} and the rising time of the Kerr dynamics. The origin of the emerging Kerr signal is assumed to arise due to the spatial overlap of the valley-polarized electron wavepacket and the probe beam. Supposing the two components exhibit a Gaussian distribution, the Kerr rotation signal θ_{Kerr} at a fixed point can be expressed as

$$\theta_{Kerr}(r) \propto n_{\mathrm{e}} \cdot I = n_{\mathrm{e},0} I_0 \left[e^{-\frac{2r^2}{R}} \right], \qquad \text{(S1)}$$

where n_e is the valley-polarized electron wavepacket, I is the probe beam profile, and R is the size (radius) of the Gaussian functions. The probe beam spot is measured to be ~ 2 μm. Here, we also assumed the two Gaussian distributions do not change significantly as a function of the pump-probe delay, and the transport speed of the valley-polarized electron wavepacket is constant. Then, the transport velocity can be obtained by dividing R by the rising time; the rising time is the temporal waist obtained from the fit to the Kerr rotation transients.

Supplementary Note 7 - Theoretical analysis based on the Kubo formula

The monolayer MoS_2 has a C_{3h} symmetry with a strong spin-orbit coupling (SOC). The Hamiltonian is

$$\hat{H} = at(vk_x\hat{\sigma}_x + k_y\hat{\sigma}_y) + \frac{\Delta}{2}\hat{\sigma}_z - \lambda v\frac{\hat{\sigma}_z - 1}{2}\hat{s}_z \ , \qquad \text{(S2)}$$

where a is the lattice constant, t is the effective hopping integral, $\mathrm{v} = \pm 1$ is the valley index, $\hat{\sigma}$ is the Pauli matrix, and Δ is the bandgap. The last term represents SOC, where 2λ and $\hat{s}_z$ denote the spin splitting of the valence band and the Pauli spin matrix, respectively. Using the cell periodic Bloch function $\boldsymbol{u}$(k), the Berry curvature is defined as

$$\Omega_n(\mathbf{k}) \equiv \hat{z} \cdot \nabla_k \times \langle u_n | i\nabla_k | u_n(\mathbf{k})\rangle, \qquad \text{(S3)}$$

and the Berry curvature in the conduction band is [10]

$$\Omega_{\mathrm{C}}(k) = -v\frac{2a^2t^2\Delta'}{[\Delta'^2 + 4a^2t^2k^2]^{\frac{3}{2}}} \ , \qquad \text{(S4)}$$

where$\Delta' = \Delta - vs_z\lambda$. Because the Berry curvature is an intrinsic property, one can isolate the intrinsic contribution from various extrinsic effects. The valley Hall velocity v_{VH} can be expressed as equation (1) using the electric field and the Berry curvature [11]. Besides, we used the approach of calculating valley Hall conductivity σ_{VH} to estimate the valley Hall mobility.

$$\sigma_{\mathrm{VH}} = -\xi\frac{e^2}{\hbar}\sum_n \frac{d\mathbf{k}}{(2\pi)^2} f(\varepsilon_n(\mathbf{k}))\Omega_{\mathrm{C}}(\mathbf{k}), \qquad \text{(S5)}$$

where ξ is the antisymmetric tensor, n is the band index in the 2D limit, $f(\varepsilon_n(\mathbf{k}))$ is Fermi-Dirac distribution, and $\Omega_{\mathrm{C}}(\mathbf{k})$ is the Berry curvature derived in equations (S3) and (S4). Based on the Berry curvature and known parameters of the monolayer MoS_2 [10], the intrinsic

contribution can be calculated. Then, the valley Hall transport mobility μ_{Hall} is derived as a function of ρ for the comparison with the experimental results.

Supplementary Note 8 – Gate voltage dependence of the valley Hall velocity

For the valley Hall mobility, we wish to note that it does not exactly mean that mobility is proportional to the amount of injected electrons. Instead, changing V_G, i.e. increase or decrease of the total electron population, leads to the changes of valley Hall mobility by rendering more or less electrons to be affected by a larger or smaller Berry curvature distribution in the momentum space.

Just to illustrate the main point, we reiterate once again that the intrinsic valley Hall velocity $\mathbf{v}_{\perp}$ depends on the Berry curvature $\Omega(\mathbf{k})$ and the electric field $\mathbf{E}$ applied to the electron in the band as shown in equation (1). As long as the longitudinal electric field is constant, which for most of the cases are done by applying DC source-drain potential gradient [4,12,15], the transverse valley Hall velocity would not be changed. This is exactly the same as our case. In our experiment, changing the electron density by V_G controls the electron distribution in the MoS_2 conduction band. The applied longitudinal electric field $\vec{E}_y$ makes the Fermi surface being tilted in momentum space, as schematically shown in Supplementary Fig. 13. After the thermalization and cooling are finished, the injected group of electrons fills the conduction band from the point marked as a black dashed line in Supplementary Fig. 13. When V_G increases, the electron population at the K (or K′) point increases, whose effect is explained as the band filling from (i) to (ii). This appears as a faster rising dynamic component in our time-resolved Kerr rotation because the non-zero Berry curvature is concentrated at the K and K′ point of the band extrema in the momentum space, i.e. the tilted Fermi surface makes the injected electrons away from the K or K′ point.

Supplementary Table 1 - Comparison of valley Hall mobility and longitudinal mobility

Mobility (cm^2 / Vs)	Reference
4,490	This work
6.86×10^{-3}	*Science* **344**, 1489-1492 (2014) (μ_{Hall}) [12]
160	*Science* **360**, 893-896 (2018) ($\mu_{\mathrm{Hall,hole}}$) [13]
1012	*Nat. Nanotechnol*. **10**, 534-540 (2015) (μ_l) [14]
~1000	*Nat. Commun.* **10**, 611 (2019) (μ_l, 2 K) [15]

Supplementary Figures

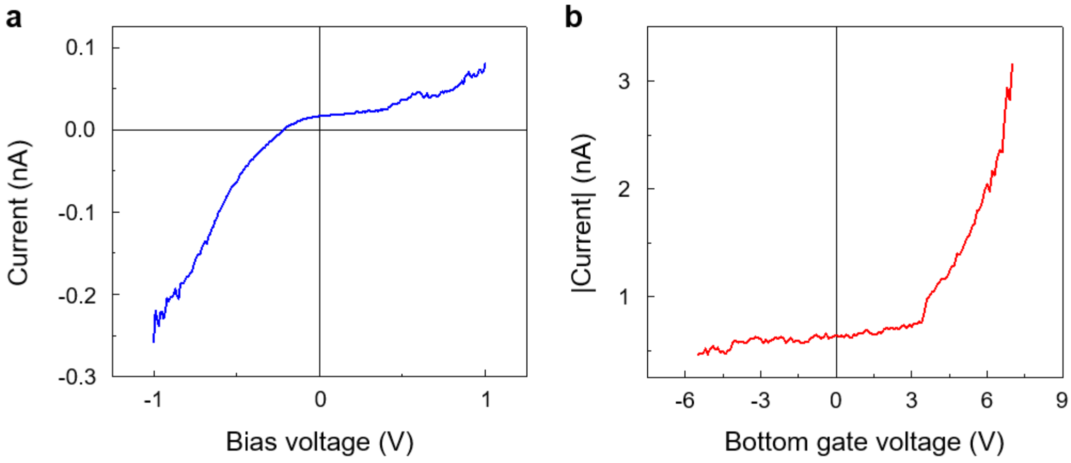

Supplementary Figure 1. Electrical characteristics of the heterobilayer device. **a.** I-V output curve of the device when the source-drain voltage is applied across the MoS_2–WTe_2 channel. **b.** V_G-dependent current through the MoS_2 channel with the source-drain bias of -1.5 V. The turn-on voltage is estimated to be 4 V.

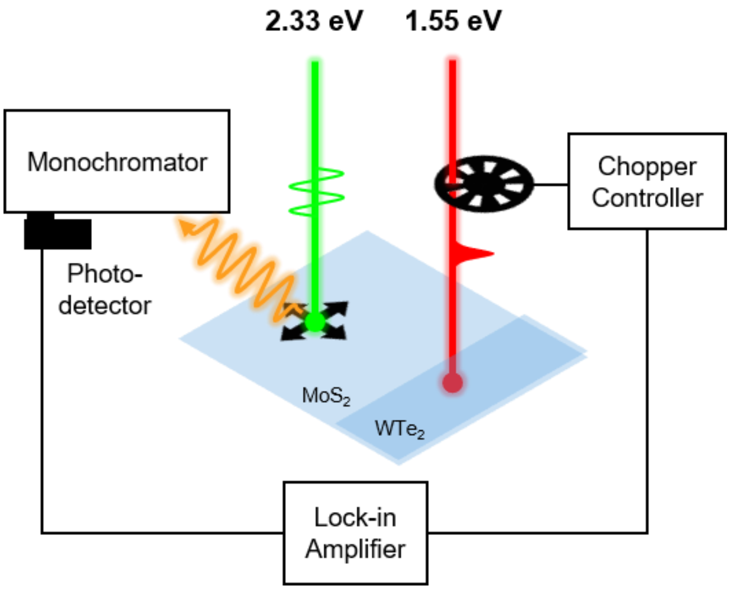

Supplementary Figure 2. A schematic diagram of the spatially resolved differential PL measurement. The change in the PL signal induced by the chopped 1.55 eV remote pump is recorded while scanning the 532 nm (2.33 eV) c.w. laser beam using Galvo scanning mirrors. The detection of the PL signal is done with a photodetector combined with the monochromator, and the change in PL according to the existence of the pump is obtained by the lock-in amplifier synchronized with the mechanical chopper.

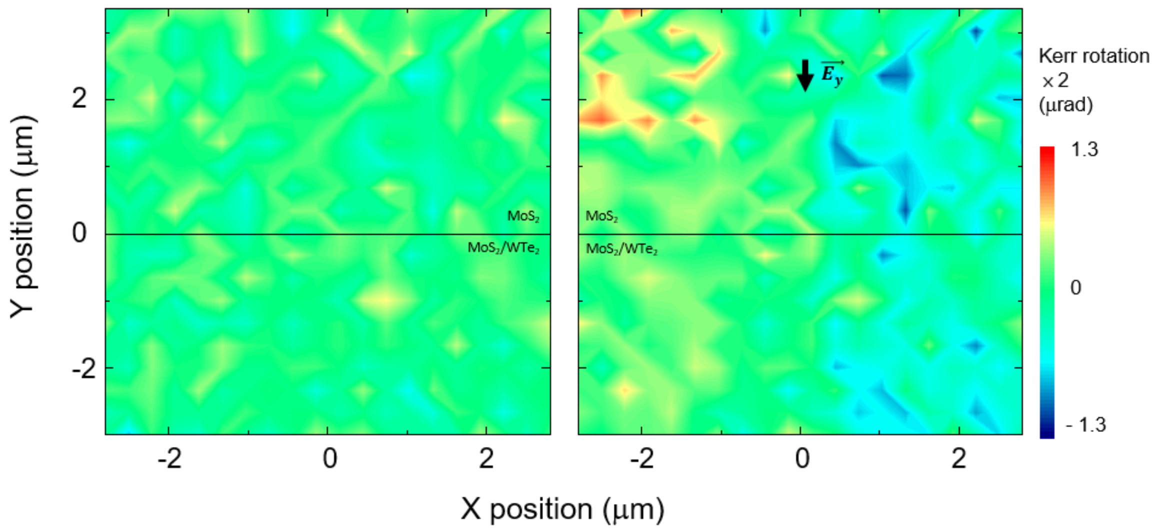

Supplementary Figure 3. The 2D map of the c.w. Kerr rotation with (right) and without (left) the longitudinal electric field. The amplitude of the Kerr rotation is doubled for further comparison with TRKR data. The relatively small valley polarization that observed all over the scanning area is distinctly different from the 2D scanning TRKR signal, which implies that the electron injection is the origin of the VHE in our TRKR experiments.

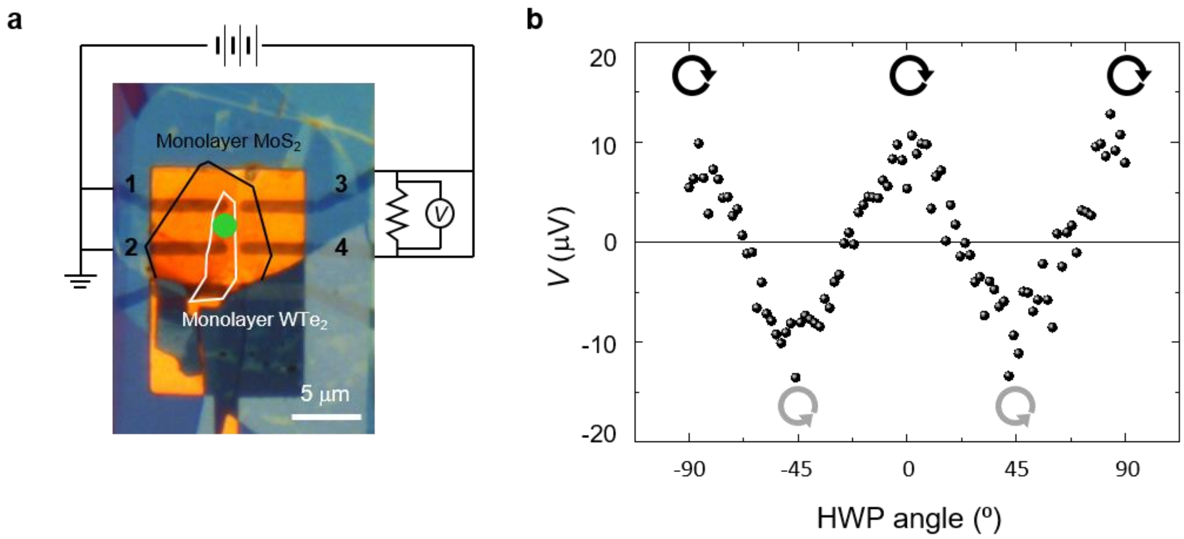

Supplementary Figure 4. a. An optical microscope image of the device used in the photocurrent measurement. The filled green circle denotes the c.w. laser with its actual spot size (2 μm). **b.** The light helicity-dependent voltage between contact 3 and 4 shows the significant sign flip of the voltage with respect to the helicity of the pump (1.55 eV) while there is no direct excitation of electrons in MoS_2. Thus, this result clearly demonstrates the existence of the VHE by the injected electrons.

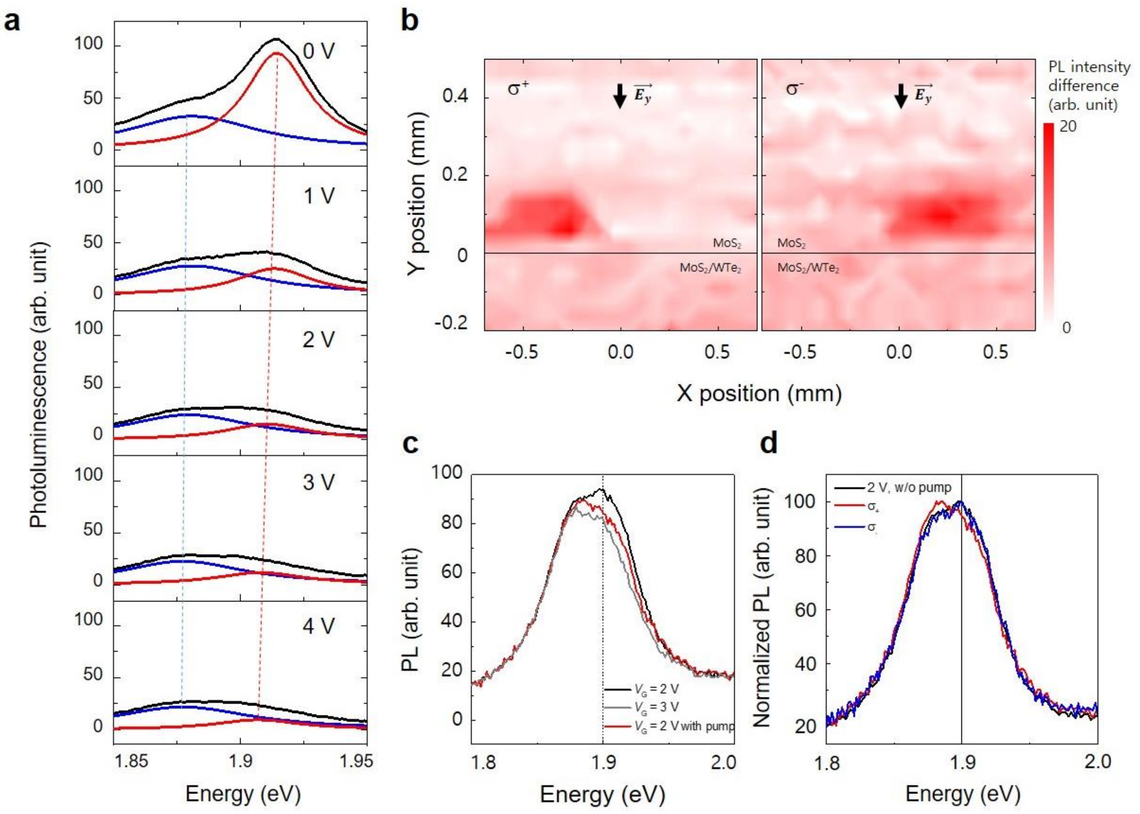

Supplementary Figure 5. a. The V_G-dependent PL of the monolayer MoS_2. Black line represents the total PL signal while the red and blue lines denote the exciton and trion component, respectively. Each component is extracted by a Lorentzian fitting **b.** The differential PL map when V_G = 2 V (same with Fig. 2a of the main text). The transverse deflection of the PL suppression reveals the injection of the spin-polarized electrons and the associated valley Hall transport. **c.** PL spectra when V_G = 2 V, V_G = 3 V, and V_G = 2 V with remote pump. **d.** Helicity-resolved PL spectra when V_G = 2 V. Compared to the PL spectrum without the remote pump (black), the injection of spin-polarized electrons change σ^+ component of the PL (red) while σ^- component of the PL (blue) remains unaffected.

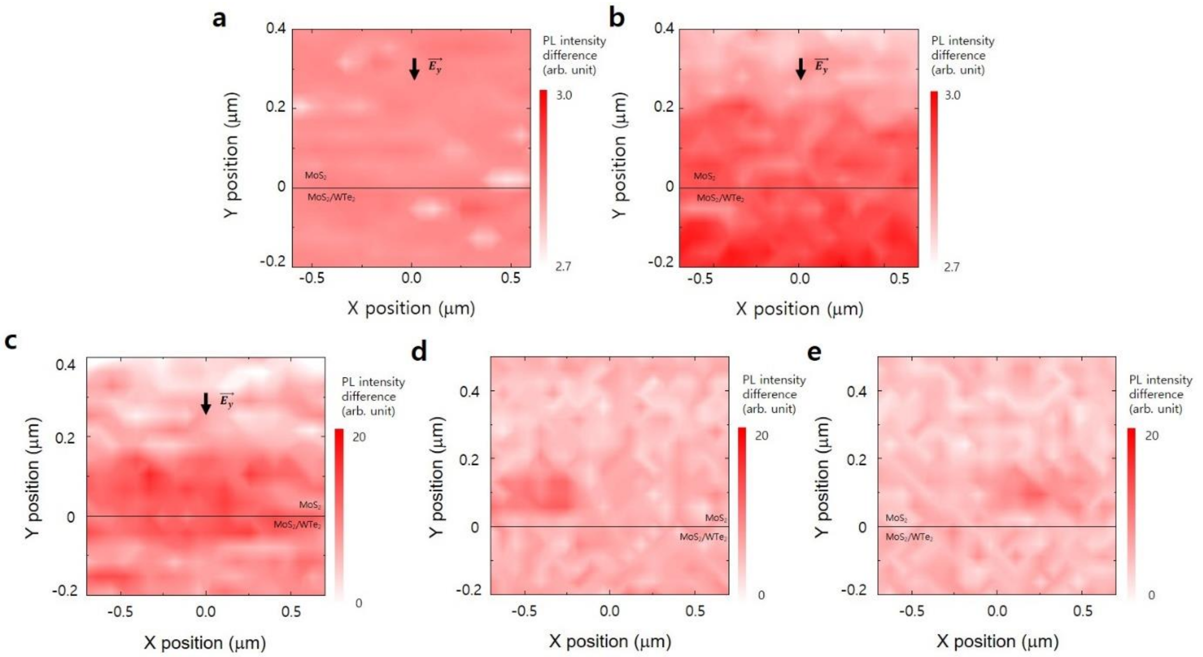

Supplementary Figure 6. Additional data for the spatially resolved differential PL, taken from device #1. The data are shown when V_G is **a.** -5 V and **b.** 6 V. **a.** When the Fermi level is sufficiently below the conduction band edge (V_G = -5 V) (see Supplementary Fig. 1(b)), the 1.55 eV pump excitation in MoS_2 has not enough energy to generate any electrons nor holes that would be injected into MoS_2. Also, due to the type 1 heterojunction between MoS_2 and WTe_2, the difference in PL is not induced, as shown in the data. **b.** When V_G = 6 V, the degenerated electron doping of the MoS_2 makes the direct excitation possible by the 1.55 eV pump. Therefore, the 1.55 eV pump no longer provides excess electrons, resulting in no PL suppression. **c.** Spatially resolved differential PL (taken from device #1, V_G = 2 V) is presented when the remote pump is linearly polarized. In the absence of the applied longitudinal electrical field, we still observe a signature of the valley Hall transport. The data were taken from device #1 when the polarization of the remote pump is **d** $\sigma+$ and **e** σ^-.

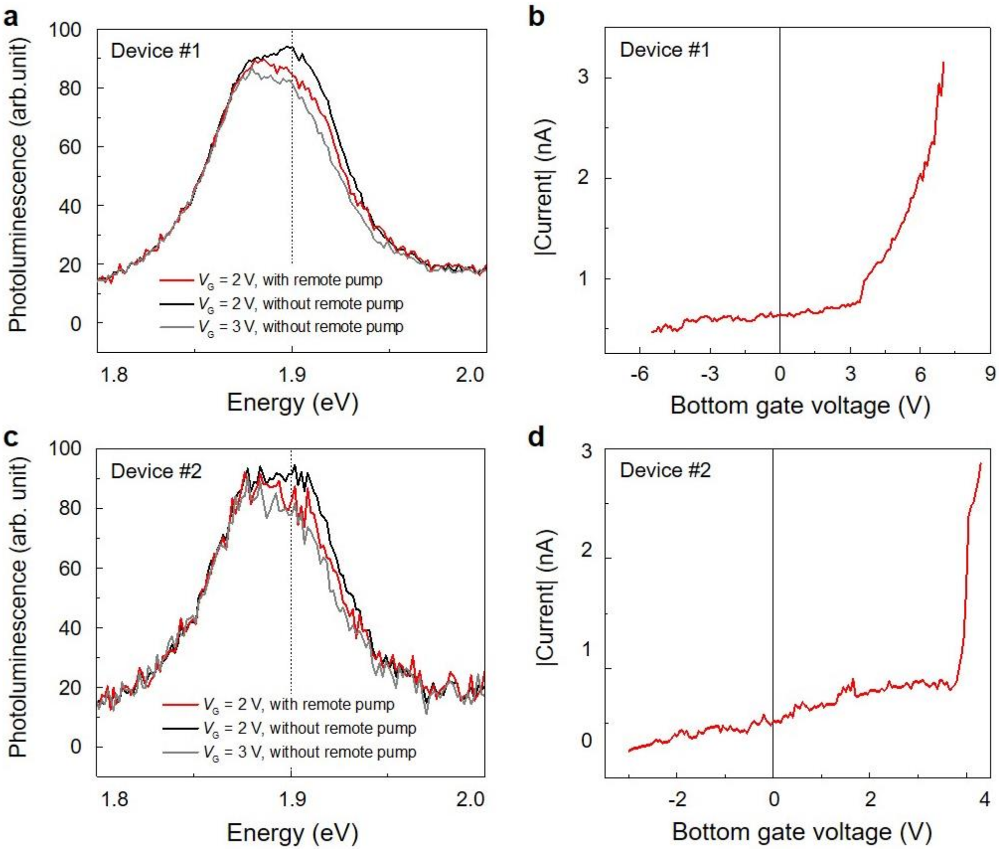

Supplementary Figure 7. Experimental results for the efficiency estimation. a. PL spectra with the remote pump when V_G = 2 V compared to the PL at V_G = 2, 3 V without the remote pump, and b. V_G-dependent transfer curve of device #1. c and d show the same experimental results for device #2.

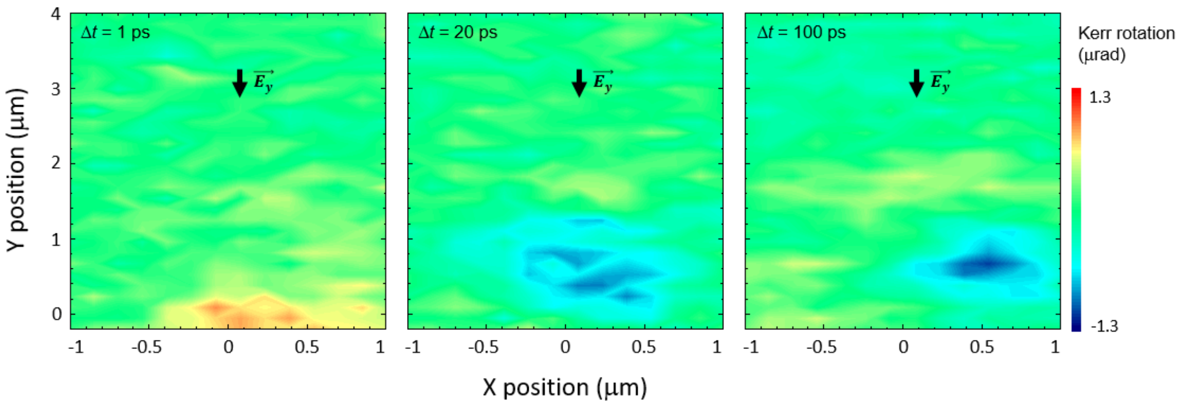

Supplementary Figure 8. The 2D scanning Kerr rotation results are shown when an opposite remote pump helicity is used. The Kerr rotation with a negative sign travels to the +x direction. Compare to the -x direction valley Hall transport shown in Fig. 3 of the main text, this results verifies the opposite valley Hall transport is induced by the opposite valley polarization.

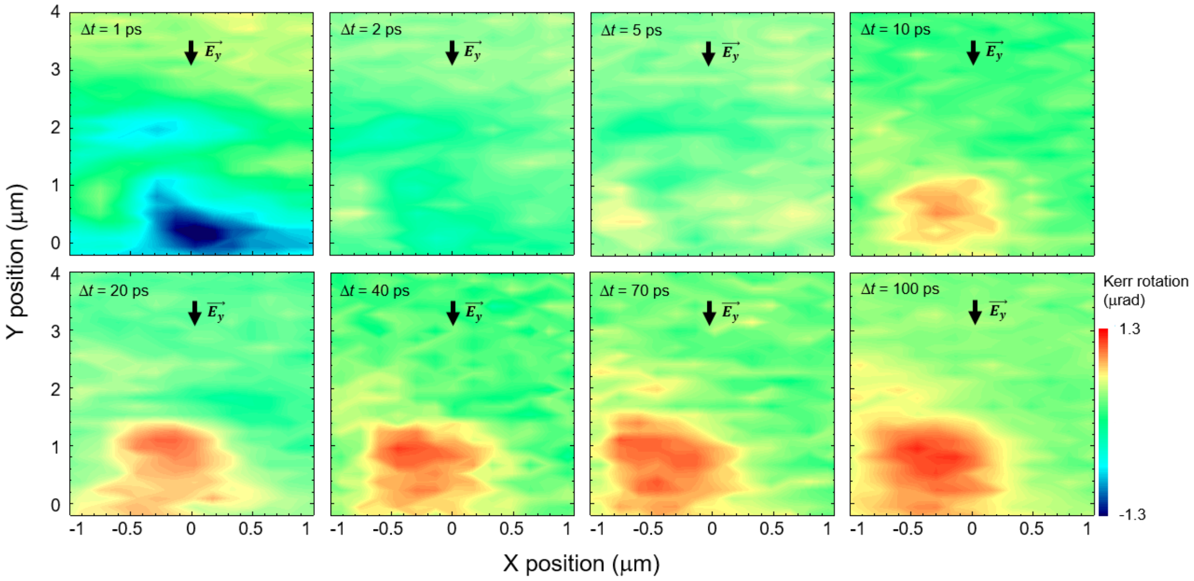

Supplementary Figure 9. Extended data of the Kerr rotation 2D map, including the 2D scanning results at Δt = 1, 2, 5, 10, 20, 40, 70, and 100 ps. Transverse transportation of the valley polarization is clearly seen.

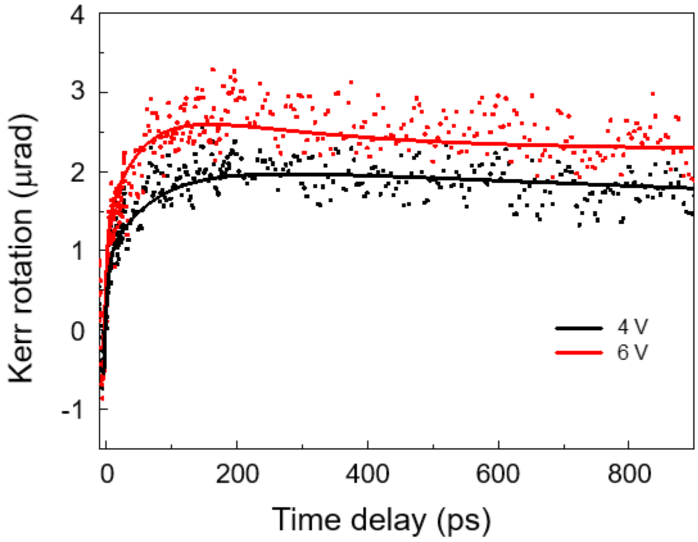

Supplementary Figure 10. Additional data for the time-resolved Kerr rotation at V_G = 4 V and 6 V. According to the V_G-dependent current measurement shown in Supplementary Fig. 1, the MoS2 is degenerately doped at V_G = 6 V. The fast decay of the valley polarization at 6 V indicates that the exciton is generated by the direct excitation of the MoS_2 conduction band electrons.

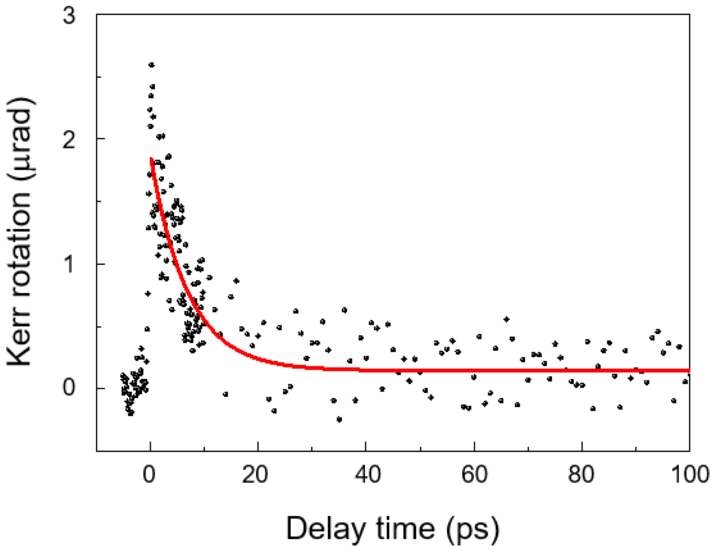

Supplementary Figure 11. The reference data of the local TRKR at the monolayer MoS_2. The circularly polarized pump (1.88 eV) and linearly polarized probe pulse (1.88 eV) are used to measure the Kerr rotation; two beams are adjusted to overlap with each other at one point within the monolayer MoS_2 area. The local TRKR data shows the decaying time constant of around 6.8 ps, which corresponds with a typical valley polarization lifetime of monolayer MoS_2 confirmed by other studies [6-9].

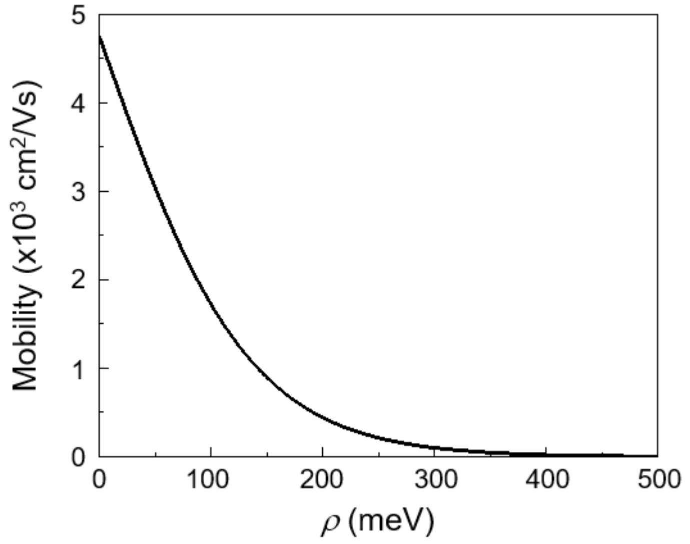

Supplementary Figure 12. Theoretical calculation of the valley Hall transport mobility based on the Kubo formula in Supplementary Note 7. The valley Hall transport mobility is calculated as a function of the energy difference between the conduction band minimum and the Fermi level. The result shows the electron mobility exceeding 4,000 cm^2/Vs near $\rho = 0$ at the intrinsic limit, which matches well with our experimental observation, as described in the main text.

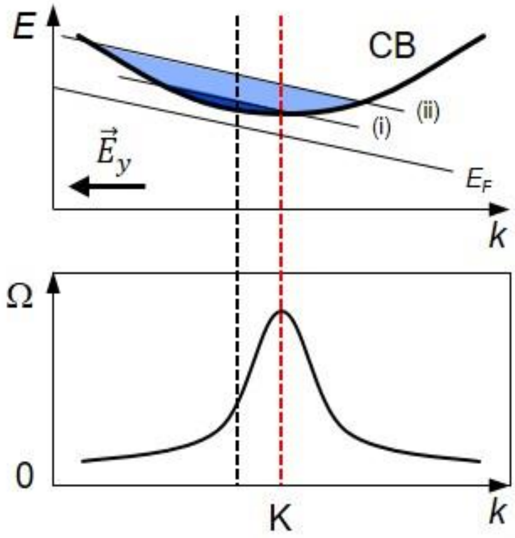

Supplementary Figure 13. Schematic diagram describing the effect of the longitudinal field ($\vec{\boldsymbol{E}}_{\boldsymbol{y}}$) on the electron distribution. K point of the momentum space is expressed as a red dashed line, and the bottom of the conduction band near the Fermi surface E_F is marked as a black dashed line. As V_G increases, the increased amount of electron injection and the higher Fermi surface lead the change of electron distribution after thermalization from (i) to (ii). Note that Berry curvature is concentrated near at K point. More electrons experience a larger Berry curvature in case (ii) compared to the case (i).

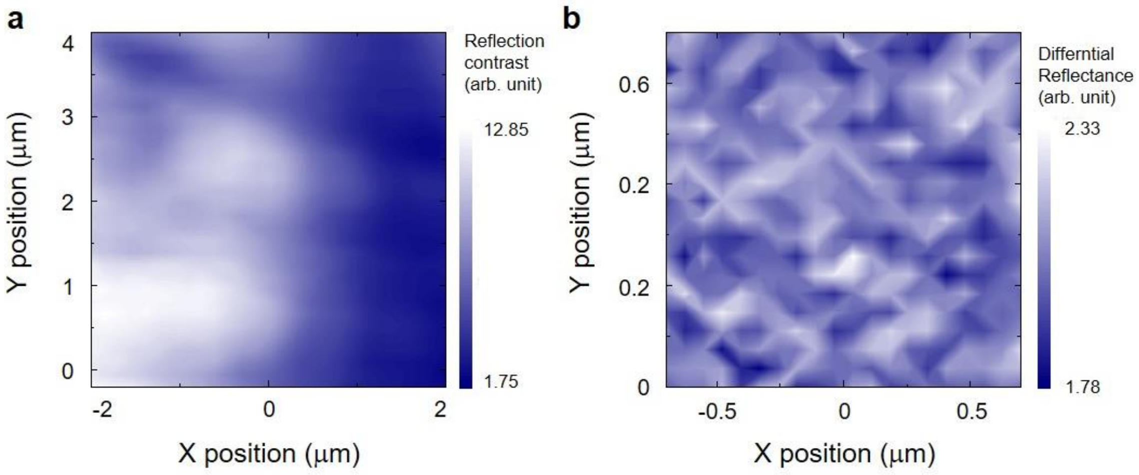

Supplementary Figure 14. Reference data from the pre-experiment measurements. **a.** The reflection contrast was measured along the region of experiments in the device to compensate the variance of local reflectance due to the microscopic inhomogeneity of the device. The amplitude of the Kerr rotation signal is recalculated by using the spatial distribution of the reflection contrast. **b.** The 2D scanning Kerr rotation microscopy was performed within the monolayer MoS_2 region (away from the WTe_2) of the same device to confirm that the unexpected excitation of electrons in MoS_2 by the low energy pump can be ruled out. When the pump (1.55 eV) is centered at (0, 0), no effect of the pump is observed when scanning the probe (1.88 eV) along the designated area. The pump-probe time delay was fixed at $\Delta t = 0$ ps.

Supplementary References

1. Tang, S., et al. Quantum spin Hall state in monolayer 1T′-WTe_2. *Nat. Phys.* **13**, 683-687 (2017).

2. Wu, S. et al. Observation of the quantum spin Hall effect up to 100 kelvin in a monolayer crystal. *Science* **359**, 76-79 (2018).

3. Rivera, P., et al. Observation of long-lived interlayer excitons in monolayer $MoSe_2$-WSe_2 heterostructures. *Nat. Commun.* **6**, 6242 (2015).

4. Lee, J., Mak, K. F., and Shan, J. Electrical control of the valley Hall effect in bilayer MoS2 transistors. *Nat. Nanotechnol*. **11**, 421-425 (2016).

5. Lee, J., Wang, Z., Xie, H., Mak, K. F., Shan, J. Valley magnetoelectricity in single-layer MoS_2. *Nat. Mater.* **16**, 887-891 (2017).

6. Xu, X., Yao, W., Xiao, D., Heinz, T. F. Spin and pseudospins in layered transition metal dichalcogenides. *Nat. Phys.* **10**, 343-350 (2014).

7. Moody, G., et al. Intrinsic homogeneous linewidth and broadening mechanisms of excitons in monolayer transition metal dichalcogenides. *Nat. Commun.* **6**, 8315 (2015).

8. Amani, M., et al. Near-unity photoluminescence quantum yield in MoS_2. *Science* **27**, 1065-1068 (2015).

9. Schaibley, J. R., et al. Valleytronics in 2D materials. *Nat. Rev. Mater.* **1**, 16055 (2016).

10. Xiao, D., Liu, G. B., Feng, W., Xu, X., Yao, W. Coupled spin and valley physics in monolayers of MoS_2 and other group VI dichalcogenides. *Phys. Rev. Lett.* **108,** 196802 (2012).

11. Nagaosa, N. et al. Anomalous Hall effect. *Rev. Mod. Phys.* **82,** 1539 (2010).

12. Mak, K. F., McGill, K. L., Park, J., McEuen, P. L., The valley Hall effect in MoS_2 transistors. *Science* **344**, 1489-1492 (2014)

13. Jin, C. et al. Imaging of pure spin-valley diffusion current in WS_2-WSe_2 heterostructures. *Science* **360**, 893-896 (2018).

14. Cui, Xu. et al. Multi-terminal transport measurements of MoS_2 using a van der Waals heterostructures device platform. *Nat. Nanotechnol.* **10**, 534-540 (2015).

15. Wu, Z. et al. Intrinsic valley Hall transport in atomically thin MoS_2. *Nat. Commun.* **10**, 1-8 (2019).
